# Supplementary figures and images for: Lineage-tracing and translatomic analysis of damage-inducible mitotic cochlear progenitors identifies candidate genes regulating regeneration
Source: PLoS Biol. 2021 Nov 10;19(11):e3001445. doi: 10.1371/journal.pbio.3001445 (PMC8608324; doi:10.1371/journal.pbio.3001445)

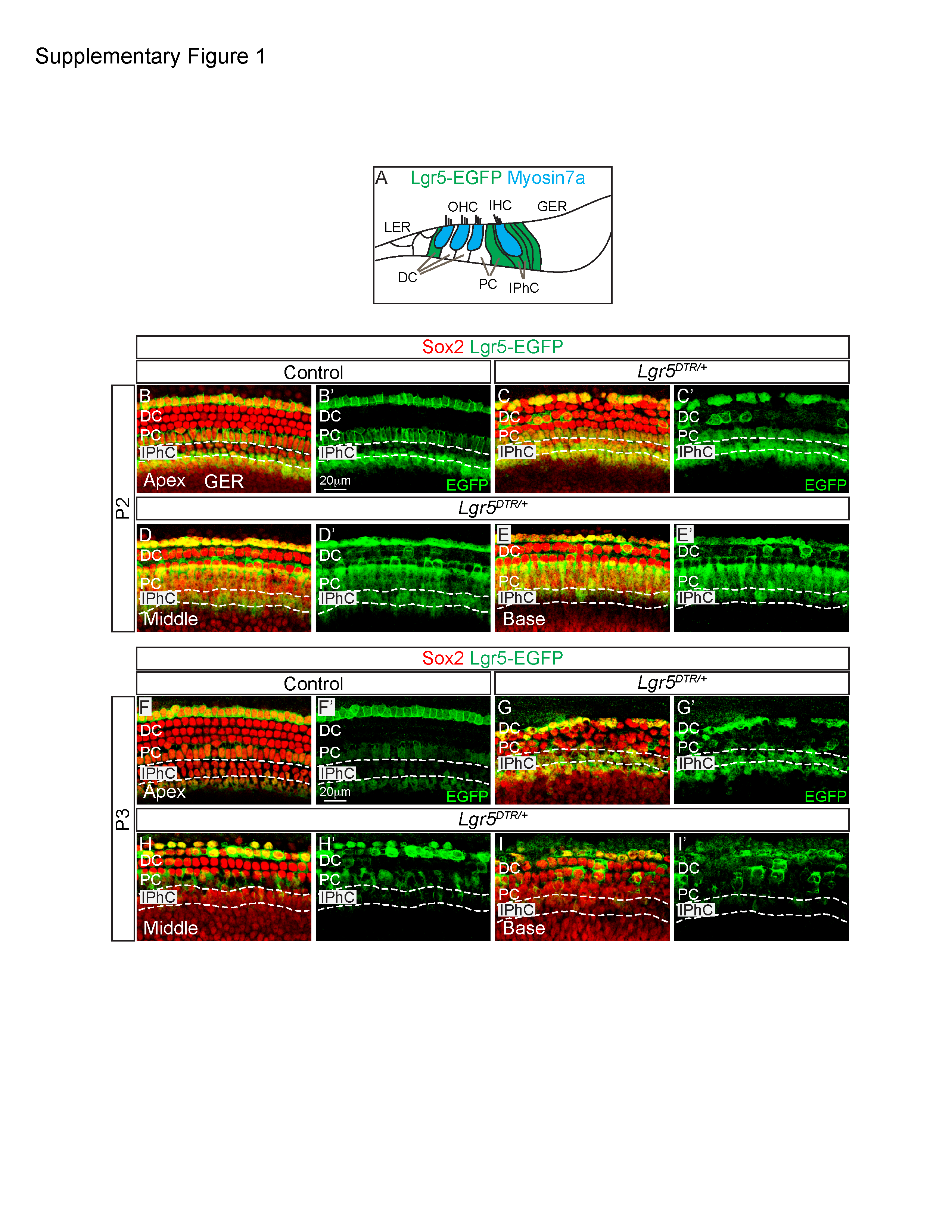

Supplement: S1 Fig — (A) Cartoon depiction of Lgr5-EGFP expression in the P1 Lgr5DTR/+ cochlea. (B, F) Undamaged, saline-treated Lgr5DTR/+ (control) cochleae showed Lgr5-EGFP expression in IPhCs, inner PCs, and the third row of DCs in the apical turn at P2 and P3. (C-E, G-I) In the DT-treated Lgr5DTR/+ cochleae, the first and second rows of DCs and outer PCs ectopically expressed Lgr5-EGFP with partial loss in all 3 turns at both ages. DC, Deiters’ cell; DT, diphtheria toxin; GER, greater epithelial ridge; IHC, inner hair cell; IPhC, inner phalangeal cell; LER, lesser epithelial ridge; OHC, outer hair cell; PC, pillar cell. (TIF) [file pbio.3001445.s001.tif]

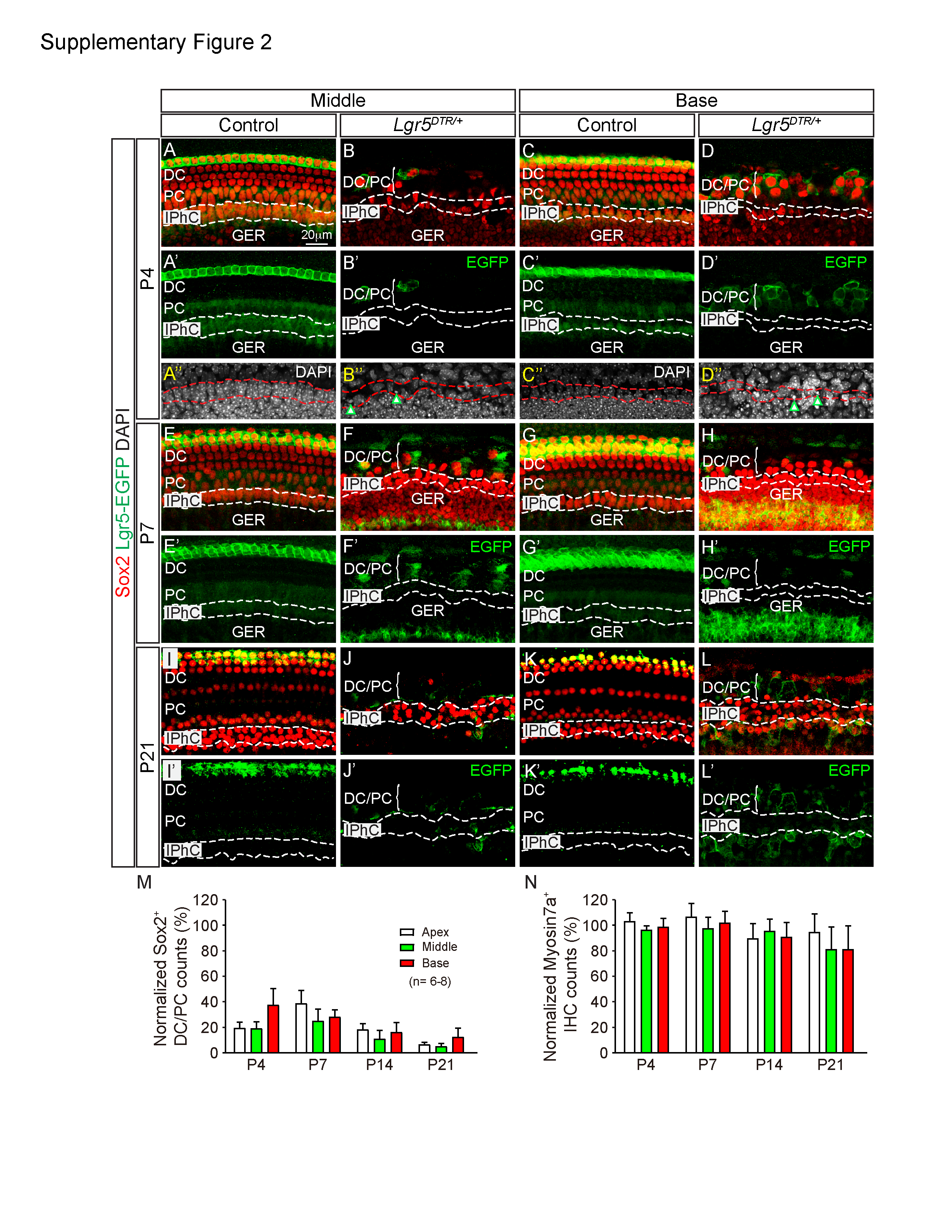

Supplement: S2 Fig — (A, C, E, G, I, K) Shown are representative confocal images from middle and basal turn of the saline-treated Lgr5DTR/+ (undamaged) cochleae. Lgr5-EGFP signals were detected in the lateral GER, IPhCs, inner PCs, and the third row of DCs at P4 and P7, while at P21, expression was restricted to the third row of DCs at P21. No pyknotic nuclei were detected at P4. (B, D) In the P4 DT-treated Lgr5DTR/+ cochlea, most Sox2+ cells were lost in the IPhC and PC/DC regions. (B”, D”) Many pyknotic nuclei were observed in the IPhC region (arrowheads). (F, H, J, L) In each turn of the P7 and P21 DT-treated Lgr5DTR/+ cochlea, IPhCs were present and at cell densities comparable to controls. However, Sox2+ SCs in the PC/DC region remained depleted. (M, N) Quantification of Sox2+ or Myosin7a+ cells (per 160 μm) in the apical, middle, or basal cochleae (normalized to control). Normalized Sox2+ PC/DC counts were reduced by P4 and partially regenerated at P7, followed by a delayed and progressive degeneration at P14 and P21 in the apical and middle turns (n = 6 at P4, n = 8 at P7, n = 6 at P14, and n = 8 at P21). In the basal turn, normalized Sox2+ PC/DC counts gradually decreased. There were no detectable changes in normalized Myosin7a+ IHC counts. Dashed lines highlight IPhC region. n = 4–8. See S1 Data for M and N. DC, Deiters’ cell; DT, diphtheria toxin; GER, greater epithelial ridge; IHC, inner hair cell; IPhC, inner phalangeal cell; PC, pillar cell; SC, supporting cell. (TIF) [file pbio.3001445.s002.tif]

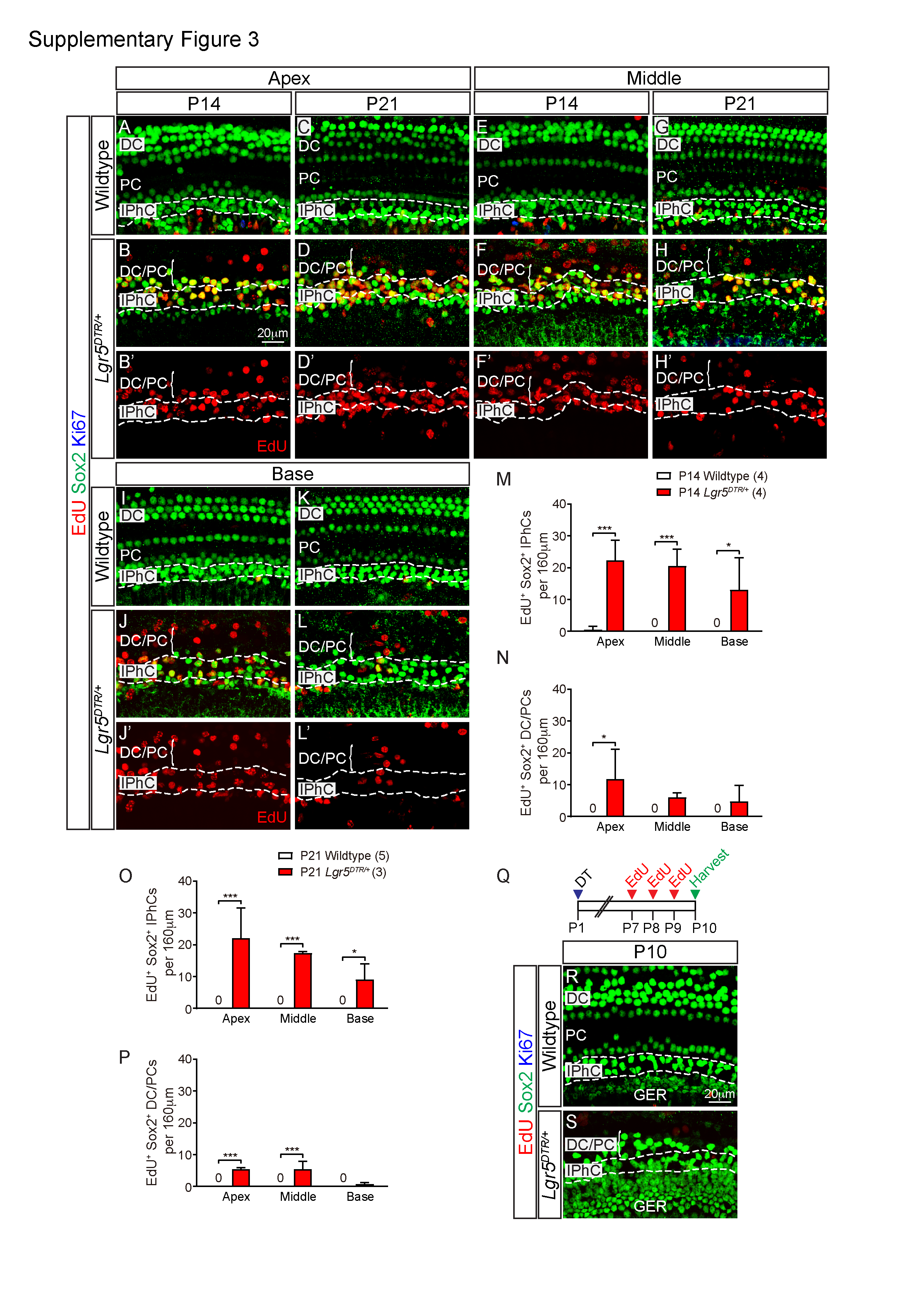

Supplement: S3 Fig — (A-L) Representative images of the apical, middle, and basal turns of DT-treated wild-type (control) undamaged cochleae showing no EdU+ or Ki67+ Sox2+ SCs at P14 or P21. The IPhC region is outlined by dashed lines. In the DT-treated P14 and P21 Lgr5DTR/+ cochlea, many EdU+ Sox2+ cells remained in IPhC region in all 3 turns. No Ki67+ Sox2+ cells were detected in the IPhC or PC/DC regions. (M-P) Quantification of EdU+ Sox2+ cells in the apical, middle, and basal turns of control (DT-treated wild type) and damaged (DT-treated Lgr5DTR/+) cochleae at P14 and P21. Wild-type cochleae had almost no EdU+ Sox2+ cells at both ages examined. Conversely, there were significantly more EdU+ Sox2+ IPhCs in the damaged cochleae with an apex-to-base gradient at both P14 and P21. Some EdU+ Sox2+ cells survived in PC-DC regions in the damaged cochleae in all 3 turns at both ages. (Q) Schematic showing DT administration to P1 wild-type or Lgr5DTR/+ mice. EdU was injected daily from P7 to P9, and cochleae were examined at P10. (R, S) In both DT-treated wild-type (control) and DT-treated Lgr5DTR/+ cochleae, there were no EdU+ or Ki67+ Sox2+ cells. Data represent mean ± SD. *p < 0.05, ***p < 0.001 (two-way ANOVA with Tukey’s multiple comparisons test). n = 3–5. See S1 Data for M-P. DC, Deiters’ cell; DT, diphtheria toxin; GER, greater epithelial ridge; IPhC, inner phalangeal cell; PC, pillar cell; SC, supporting cell. (TIF) [file pbio.3001445.s003.tif]

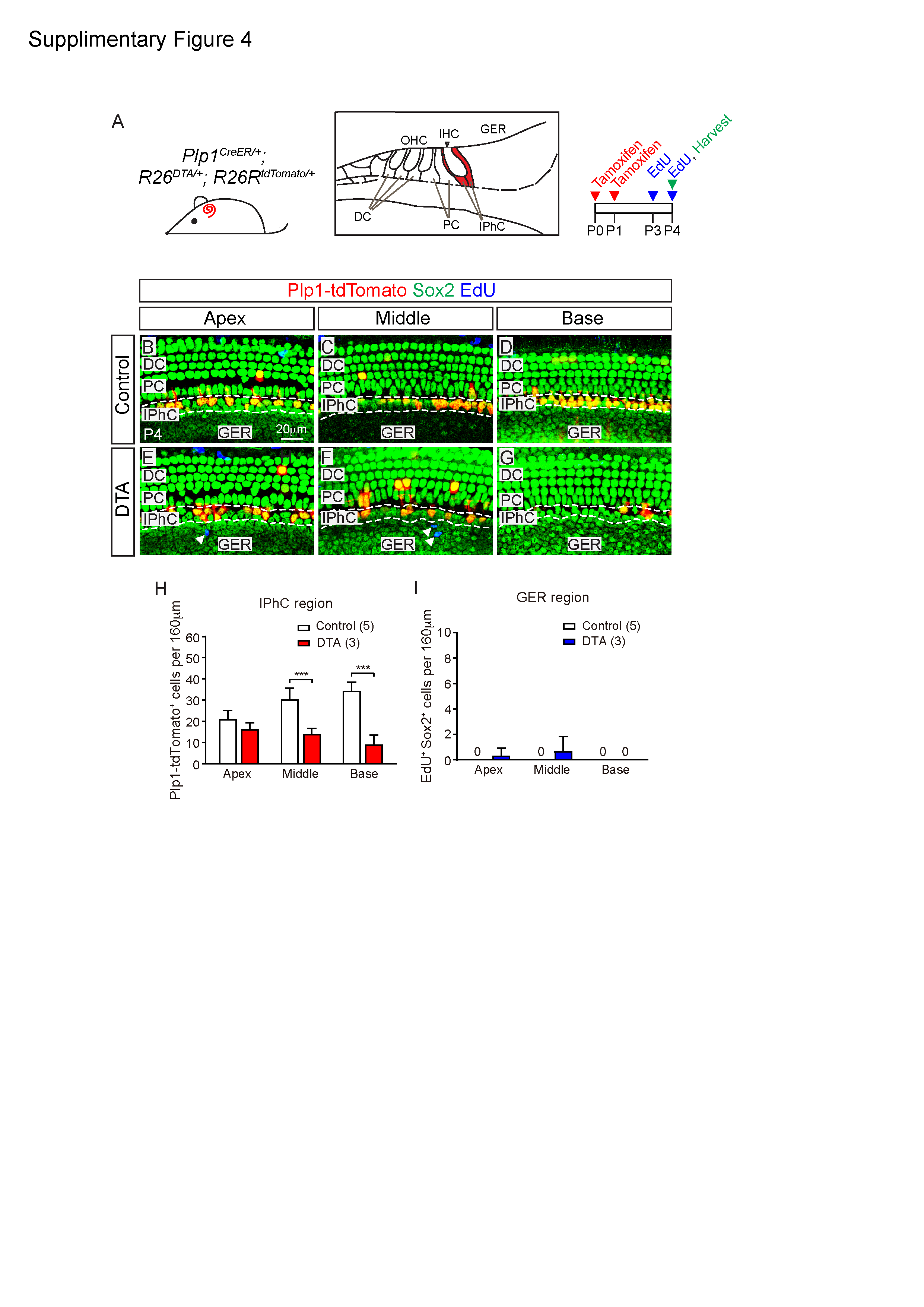

Supplement: S4 Fig — (A) Tamoxifen was injected into P0 and P1 Plp1CreERT/+; R26RtdTomato/+; R26RDTA/+ mice. Plp1CreERT/+; R26RtdTomato/+ mice served as undamaged controls. EdU was injected daily from P3 to P4, and cochleae were examined at P4. (B-D) Representative images of the each turn of control cochleae showing Plp1-tdTomato+ Sox2+ IPhCs (dashed lines). No EdU-labeled Sox2+ cells were detected. (E-G) In damaged cochleae, fewer Plp1-tdTomato+ Sox2+ IPhCs were detected, and rare EdU+ Sox2+ cells were detected in the GER (arrowheads) in the apical and middle turns. (H) Quantification showing a significant reduction of Plp1-tdTomato+ in the middle and basal turns. (I) EdU+ Sox2+ cells in the GER were rarely found, and the number was not significantly different from control cochleae. Data represent mean ± SD. ***p < 0.001 (two-way ANOVA with Tukey’s multiple comparisons test). n = 3–5. See S1 Data for H and I. DC, Deiters’ cell; GER, greater epithelial ridge; IHC, inner hair cell; IPhC, inner phalangeal cell; OHC, outer hair cell; PC, pillar cell. (TIF) [file pbio.3001445.s004.tif]

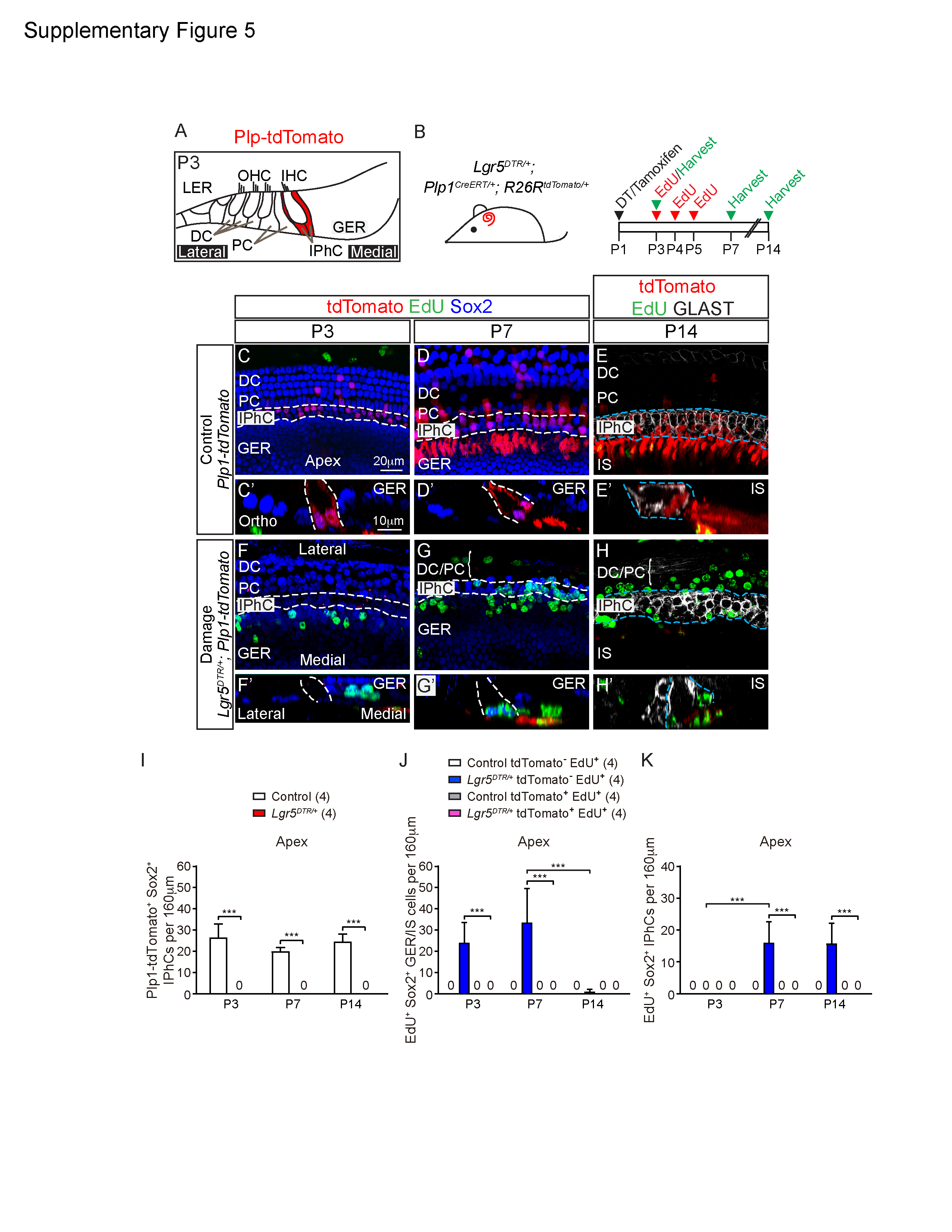

Supplement: S5 Fig — (A) Plp1-tdTomato expression in the P3 Plp1CreERT/+; R26RtdTomato/+ cochlea. (B) Schematic of the experimental paradigm: DT and tamoxifen were injected into the P1 Plp1CreERT/+; R26RtdTomato/+ (control) or Lgr5DTR/+; Sox2CreERT2/+; R26RtdTomato/+ (damage) mice. EdU was injected daily from P3 to P5, and cochleae were examined at P3, P7, or P14. (C-E) Representative images of the apical turn of control cochleae showing Plp1-tdTomato+ Sox2+ or SCs at P3 and P7. At P14, Plp1-tdTomato+ IPhCs expressed GLAST. IPhC region outlined by dashed lines. (F-H) In damaged cochleae, Plp1-tdTomato+ Sox2+ cells were not detected in the IPhC region or in the GER at any age. Conversely, EdU+ Sox2+ Plp1-tdTomato-negative cells were detected in the GER at P3. At P7 and P14, EdU+ Sox2+ Plp1-tdTomato+ cells were not found in the GER/IS or IPhC regions. Many EdU+ Sox2+ Plp1-tdTomato-negative cells were detected in the IPhC region at P7 and P14. Orthogonal views shown in C’-H’. (I) Quantification of Plp1-tdTomato+ Sox2+ SCs in the apical turn. (J, K) Quantification of EdU+ Sox2+ SCs in the apical turn. In damaged cochleae, there were no Plp1-tdTomato+ Sox2+ SCs in the GER or in the IPhC region at any age. There was, however, an increase in Sox2+ EdU+ Plp1-tdTomato-negative cells in the GER peaking at P7, followed by a reduction at P14. There were no Sox2+ EdU+ Plp1-tdTomato-negative cells in the IPhC region at P3, but many at P7 and P14. Data represent mean ± S.D. ***p < 0.001 (two-way ANOVA with Tukey’s multiple comparisons test). n = 4. See S1 Data for I-K. DC, Deiters’ cell; DT, diphtheria toxin; GER, greater epithelial ridge; IHC, inner hair cell; IPhC, inner phalangeal cell; IS, inner sulcus; LER, lesser epithelial ridge; OHC, outer hair cell; PC, pillar cell; SC, supporting cell. (TIF) [file pbio.3001445.s005.tif]

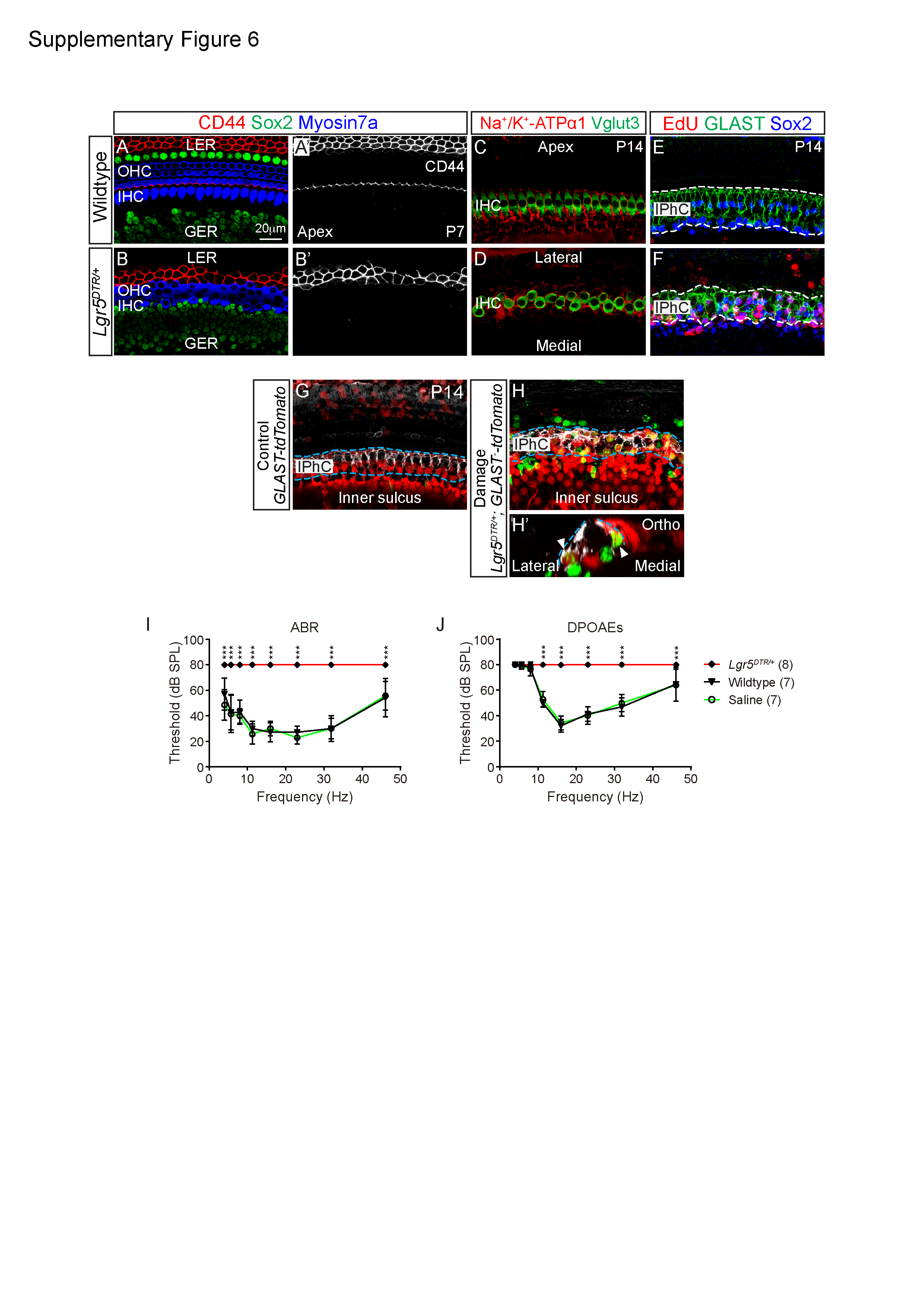

Supplement: S6 Fig — (A) In undamaged P7 (control) cochleae, CD44 is expressed in outer PCs, Claudius cells, and the LER. Representative images of the apical turn are shown. IPhC region is labeled by dashed lines. (B) In the P7 DT-treated Lgr5DTR/+ cochlea, Sox2+ SCs in the PC/DC region have degenerated, with CD44+ Claudius cells and LER appearing grossly intact. (C, D) In both the undamaged and damaged P14 cochlea, Vglut3+ IHCs were surrounded by Na+/K+ ATPase α-1-expressing, Sox2+ IPhCs. (E) GLAST expression (membranous) of Sox2+ IPhCs in the P14 undamaged cochleae. IPhC region is outlined by dashed lines. (F) In the P14 damaged cochlea, all IPhCs expressed GLAST, and most were EdU-labeled. (G) In the P14 control cochlea, there were no EdU+ GLAST-tdTomato+ SCs in the inner sulcus. GLAST+ IPhCs were mostly GLAST-tdTomato+ but not EdU+. (H) In the P14 damage cochlea, many EdU+ GLAST-tdTomato+ GLAST+ IPhCs were detected. Orthogonal view shown in H’. (I, J) P21 DT-treated Lgr5DTR/+ mice had higher ABRs and DPOAEs thresholds at all frequencies than controls. Data represent mean ± S.D. ***p < 0.001 (two-way ANOVA with Tukey’s multiple comparisons test). n = 7–8. See S1 Data for I and J. ABR, auditory brainstem response; DC, Deiters’ cell; DPOAE, distortion product otoacoustic emission; DT, diphtheria toxin; GER, greater epithelial ridge; IHC, inner hair cell; IPhC, inner phalangeal cell; LER, lesser epithelial ridge; OHC, outer hair cell; PC, pillar cell; SC, supporting cell; SPL, sound pressure level. (TIF) [file pbio.3001445.s006.tif]

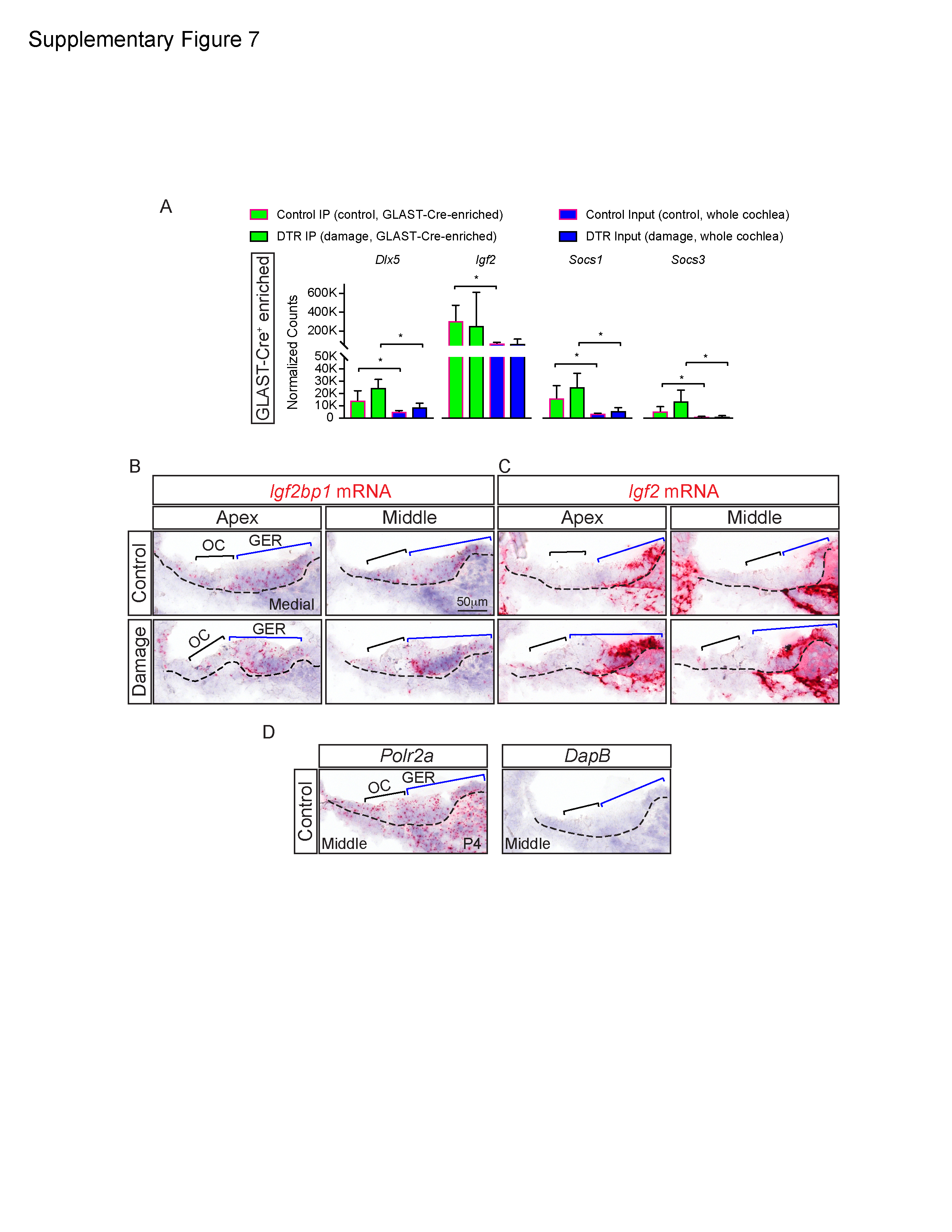

Supplement: S7 Fig — (A) Validation of a selection of genes (Dlx5, Igf2, Socs1, and Socs3) enriched in GLAST-Cre+ samples using nCounter. (B, C) In situ hybridization showing Igf2bp1 and Igf2 expression in the GER of control and damage P4 cochlea from the apical and middle turns. (D) Positive (Polra) and negative (DapB) controls shown. GER (blue bracket); OC (black bracket). Data represent mean ± SD. *p < 0.05 (Student t test). See S1 Data for A. DTR, diphtheria toxin receptor; GER, greater epithelial ridge; IP, immunoprecipitation; OR, organ of Corti. (TIF) [file pbio.3001445.s007.tif]

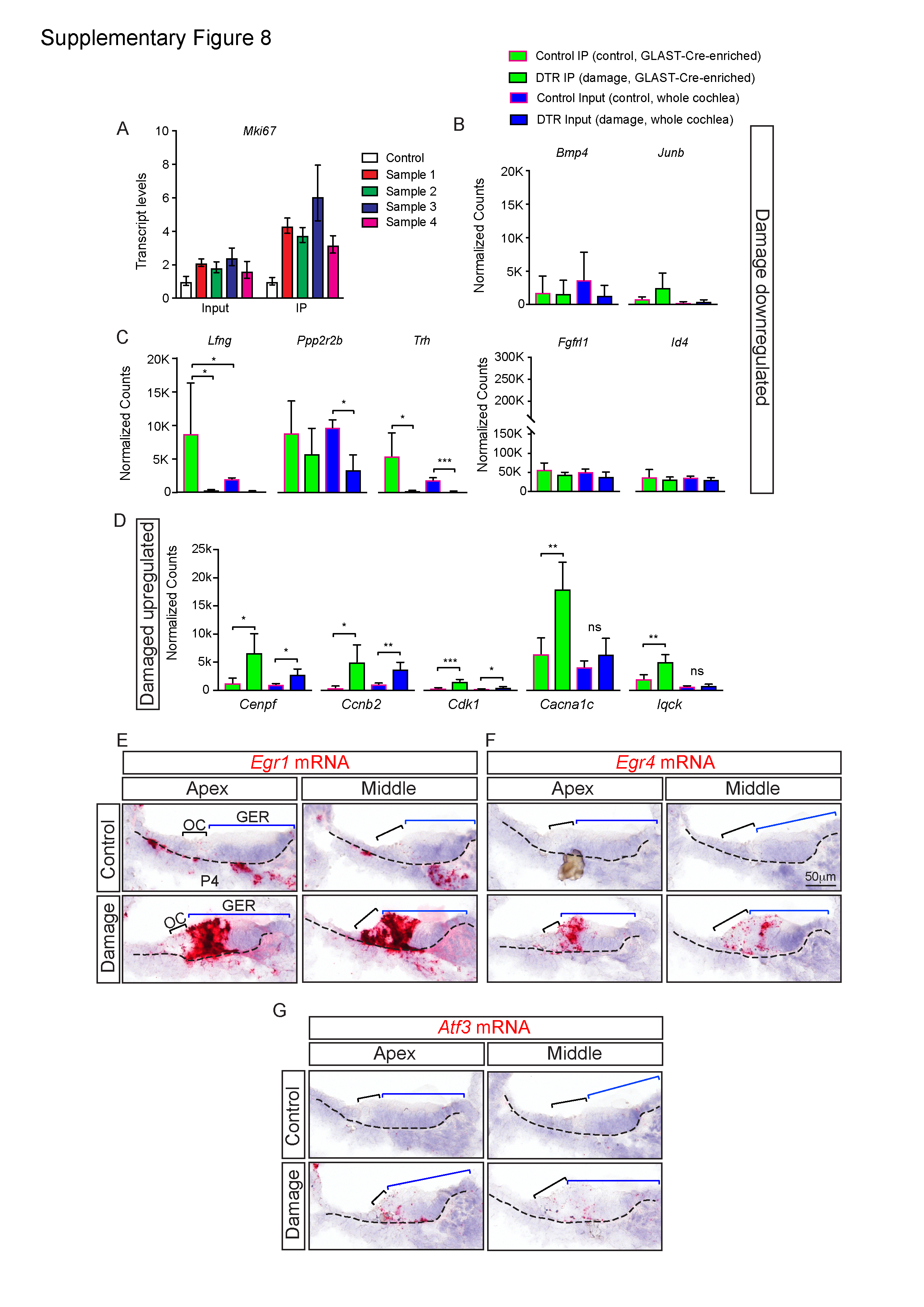

Supplement: S8 Fig — (A) qPCR showing MKi67 mRNA expression from individual control and DTR samples. (B-D) nCounter was used to valid another 12 DEGs as a result of damage. Eight were successfully validated (Lfng, Ppp2r2b, Trh, Cenpf, Ccnb2, Cdk1, Cacna1c, and Iqck), and 4 (Bmp4, Fgfrl1, Junb, and Id4) were tested and not validated. (E-G) Egr1, Egr4, Atf3 mRNA expression was minimally expressed in the undamaged cochleae. Expression was robust in the lateral GER and OC after damage. Shown are sections from the apical and middle turns. GER (blue bracket); OC (black bracket); data represent mean ± SD. *p < 0.05, **p < 0.01, ***p < 0.001 (Student t test). See S1 Data for A-D. DEG, differentially expressed gene; DTR, diphtheria toxin receptor; GER, greater epithelial ridge; IP, immunoprecipitation; OR, organ of Corti. (TIF) [file pbio.3001445.s008.tif]

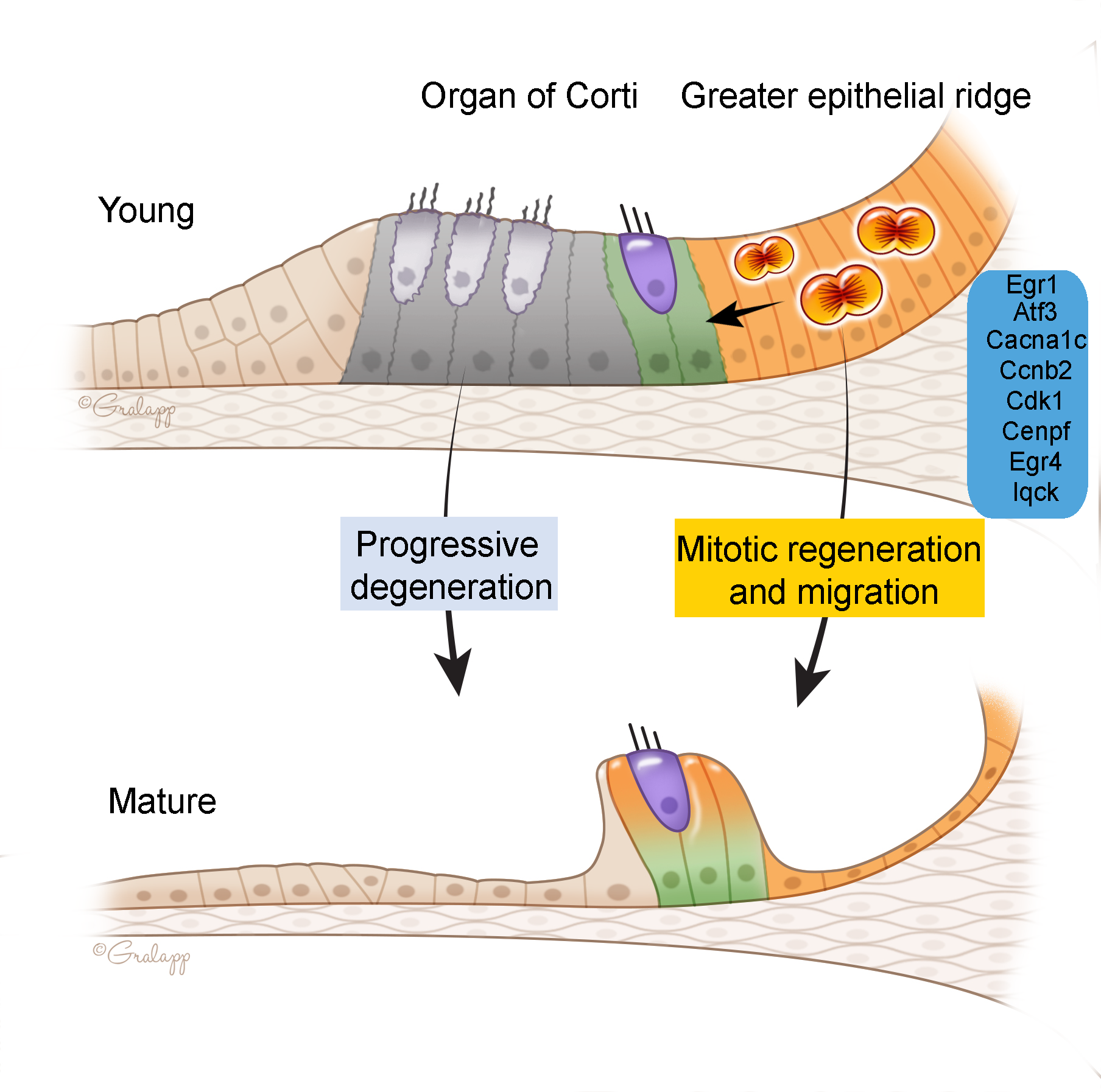

Supplement: S9 Fig — (TIF) [file pbio.3001445.s009.tif]
