# Supplementary material for: Lineage-tracing and translatomic analysis of damage-inducible mitotic cochlear progenitors identifies candidate genes regulating regeneration
Source: PLoS Biol. 2021 Nov 10;19(11):e3001445. doi: 10.1371/journal.pbio.3001445 (PMC8608324; doi:10.1371/journal.pbio.3001445)
Supplement: Attachment — Submitted filename: Pbio point by pointV4.docx [file pbio.3001445.s027.docx]

Reviewer #1:

The authors perform a study to selectively ablate Lgr5+ cells in the neonatal cochlea. Lgr5+ in the GER was initially thought to be responsible for maintaining stemness in the post-mitotic cochlea; however, the authors now describe a different population of cells in the GER contribute to regeneration. The idea that the GER holds some potential to proliferate has been known for a while and is not novel though. The mechanisms that regulate this stemness has yet to be unveiled. The authors use Lgr5-DTR to study spontaneous regeneration (has been demonstrated before in several studies) of IPhCs from the Lgr5- cell population in the GER. The study is well-done but there is quite a bit of distracting data/ claims that are unresolved, difficult to consolidate and take away from the main crux of the story. As a result, the manuscript, as written, feels descriptive without functional testing of these ideas.

Thank you for the feedback. We have revised the manuscript to clarify the points made to address your comments.

Major points:
1. Loss of OHCs, but only 3rd DC has Lgr5. Is Lgr5-DTR too toxic that it's killing neighboring cells? Some explanation of why other cells in the lateral compartment are affected, is warranted.
This is an excellent point. Even though Lgr5-GFP expression is limited to inner pillar cells, the 3^rd^ row of Deiters’ cells and inner phalangeal cells in P1 Lgr5-DTR mice, DT leads to degeneration of most supporting cells in the lateral compartment. This expression pattern is consistent with scRNA seq data (Kolla et al 2020) and *in situ* data (Chai et al., JARO 2012). However, in new data provided (Figure S1) after P1 DT injection, we found that Lgr5 becomes expressed in the first and second rows of Deiters’ cells and outer pillar cells in P2 and P3 Lgr5-DTR mice, but not in the undamaged controls. Even though diphtheria toxin’s half-life has been estimated to be 7.5hrs in other tissues (Yamaizumi PNAS 1982), it is possible that DT remains active in the inner ear and exerts toxicity on supporting cells in the lateral compartment. Alternatively, degeneration of the third row of Deiters’ cells can lead to loss­ of other supporting cells and OHC in a non-cell autonomous fashion. We have added these 2 points in the discussion section (p.21).

2. Figure 1- IHCs look impacted both in number and morphology. Do any of the HCs arise from the proliferated cells from the GER? i.e,. EdU positive?
We have not observed any EdU-positive or fate-mapping IHCs in any GlastCre-tdTomato-Lgr5-DTR or Lgr5-DTR mice (P3-P21), suggesting no mitotically or GER-derived inner hair cells. The IHC counts in Lgr5-DTR mice were also normal and comparable to controls at several timepoints (P3-P21), we therefore conclude that they are surviving and not regenerated IHCs, whose morphology has changed because of degeneration of surrounding inner phalangeal cells.

3. Figures 1E"-I". What am I supposed to take away from these images? Not described in the text.
We apologize for this confusion. These are images (now Fig. 1K-N) to illustrate 1) loss of inner phalangeal cells 2) degeneration of supporting cells in the lateral compartment, 3) regeneration of IPhCs at P7 (G’), 4) delayed OHC loss and survival of IHC, and 5) survival of regenerated IPhCs at P14 and P21 (I’ and K’). We have added the description now to the results section.

4. Figure 1L there are fewer EdU positive cells at P7, P14 and P21 than at P4, suggesting that they died off. But the authors say that they survive. Image does not support this statement.
We think that the reviewer is referring to Figure 2K, where a graph of Edu^+^ Sox2^+^ cells in the GER is shown. This graph has now been removed to avoid confusion. The reviewer is correct that some EdU^+^ cells in the GER died off, which may be in part due to the normal apoptotic process of the GER (see point #14). However, we observed an increase in EdU^+^ inner phalangeal cells in all three cochlear turns that remained present from P7 to P21 (Figure 2L-N, S3, Table S4), suggesting that some of the GER EdU^+^ cells migrated to replenish inner phalangeal cells and subsequently survive. As controls, no inner phalangeal cells in the control cochlea were EdU-labeled at all ages examined.

We now show counts of Edu^+^ IPhCs from all three cochlear turns. To verify that Edu^+^ IPhCs originated from the GER, we have performed concurrent mitotic and fate-mapping (shown in Figure 3, also see points #7).

5. Figure 2A- Very difficult to make out Edu and Ki67. Use different color scheme. Preferably, one that's colorblind friendly.
We apologize for this. We have revised figure 2 and added single channel images for EdU and Ki67 to more clearly show that it is increased first in the GER, then in the inner phalangeal cell region (EdU^+^ cells) in the Lgr5-DTR cochlea.

6. Figure 3- Pax2Cre; Lgr5-DTR, Atoh1-mCherry, Rosa26R-GCaMP3. If Lgr5 cells die off as early as E8.5 with Pax2-Cre, how is there a cochlea at all?

Even though the Lgr5-DTR allele is present at E8.5, DT was administered postnatally at P1. Therefore, Lgr5^+^ cells should not die early on, but only after DT injection in the postnatal period.

7. Figure 5- What is E'-J' supposed to tell me? No explanation.
We apologize for this. E’-J’ are orthogonal images to illustrate the fate-mapping data in E-J, where tdTomato^+^ EdU^+^ GER cells were observed to migrate into the IPhC region surrounding the IHCs and express the IPhC marker GLAST. This supports point #4.

Per Reviewer 3’s suggestion, we have moved the P3 and P7 data to Figure 3, and P21 data to Figure 5. We have also added description of these orthogonal images to their respective figure legends.

8. Page 12, line 7: 'Collectively, these results suggest that regenerated IPhCs are partially mature/functional and remained viable in the mature organ.' I am not sure if the authors can make this conclusion.
We have removed this sentence.

9. Authors present a number of associated changes that I am not sure how they fit in: Ca2+ transients- does this relate to regeneration of IPhCs? Don't large Ca2+ waves suggest damage as opposed to any regenerative response? No functional data to test a proliferative response. Loss of Pannexin and Connexin is likely a consequence of losing cells. The authors suggest that they partake in proliferation in the GER, but there's no data to support this. Other than showing that there are associated changes, their role in regeneration is not well supported.

These are excellent points. We agree that the calcium transients can be associated with damage response and do not necessarily play a role in the proliferative response and have added these points to the discussion section (p. 20). The loss of pannexin and connexin may also be caused by supporting cell loss and these data have now been removed to avoid confusion.

10. Neurite innervation and reduced CtBP2 synapses- I only see damage. Not sure what I am supposed to make of this data and how it related to the main story of IPhC regeneration from GER. It doesn't add very much to the paper or its hypotheses.

This data is to support that IHCs survive and remain innervated. In light of the comments by Reviewers 2 and 3, we have kept this data and added a graph of synapse counts in IHCs to figure 5.

11. The authors find a number of genes that were differentially regulated post DTR but does not mean they are associated with the limited regeneration of IPhCs. None were tested. How relevant they are, are yet to be determined at this stage. Thus, making the paper descriptive in nature.

We believe that descriptive and hypotheses generating studies are also an important part of the literature. Our study describes gene changes associated with damage and regeneration, deemed novel by both Reviewers #2 and 3, and can generate many testable hypotheses. Thus, this dataset is a valuable resource to be shared via the gEAR portal and can serve as the foundation for other studies testing the roles of individual damage-responsive genes.

Minor points:
12. Figure 1A- Green Lgr5 is difficult to see. Show monochromatic image for Lgr5.
We have added single channel images to illustrate Lgr5 expression in Figure 1.

13. Page 13, line 20: P3 Sox2-tdTomato-DTR cochleae.
Is this Lgr5-DTR?
This is correct, Sox2-tdTomato-DTR cochleae contains the alleles, Sox2CreERT2, Rosa26-tdTomato, and Lgr5-DTR. We have removed the Sox2-Cre data per Reviewer #2’s recommendation.

14. Page 14, line 5: "…..Sox2-expressing cells in the GER serve as mitotic progenitors capable of replenishing lost IPhCs." Only up to a certain point though. Regenerative potential is lost after apoptosis in the GER.
This reviewer is correct that the GER is a transient structure in the neonatal cochlea. The Sox2^+^ GER degenerates between P7-10 and the area becomes occupied by Sox2-negative cells of the inner sulcus by P21. We have clarified this in the results section with the addition of “in the neonatal period” (Page 10, L11).

Reviewer #2:

The manuscript by Udagawa et al., describes the regeneration of one subtype of SCs (inner phalangeal cells) after several SC subtypes were killed using the Lgr5-DTR mouse model and injection of DT at P1. Killing of Lgr5+ SCs triggered a mitotic response in the GER that was dependent on the number of SCs killed. Fate-mapping and EdU experiments demonstrated that regenerated inner phalangeal cells were derived from the GER cells. Regenerated cells expressed markers of inner phalangeal cells, appeared functional since they displayed calcium transients, and survived into adulthood. Using the RiboTag system with a CreER specific to the GER cells, the authors also report gene clusters that were altered in different ways after damage.

Overall the manuscript is well written and the experiments are sound with appropriate controls. While this work greatly expands upon what was known previously, I don't think the paper gives enough credit to findings made by Mellado Lagarde et al., 2014 which also concluded that the GER cells were the source of the regenerated inner phalangeal cells. There are also several points that need clarification. See below for major and minor comments.

Thank you for your kind words. We have revised the manuscript as recommended.

1) Previous work by Mellado Lagarde et al., 2014 is cited however I don't think that enough credit is given to this paper.  Mellado Lagarde et al., used 3 different CreER lines to express DTA,  killing inner phalangeal cells, border cells, and in some cases also GER cells. They demonstrated that inner phalangeal cells were regenerated from neighboring GER cells (while fate-mapping was not done, the conclusion was still supported by other experiments). In addition, one of the models they used was Lgr5-CreER, which should give the same result as the Lgr5-DTR model used here. There are still many unique findings in this new manuscript (the dose response of SC death, calcium transient recordings, the fate-mapping, and of course the gene expression analysis) that make this work significant and novel. It's just not the 1st to identify GER cells as progenitors, thus, I suggest changing how the study is introduced in the Abstract and Introduction and rewriting the first and last
paragraph of the Discussion to focus on the novel findings in this study.

Thank you for pointing this out. We completely agree that it is important to highlight the work by Mellado Lagarde and colleagues on which our study builds. We have revised the abstract, introduction and discussion to better highlight their work. Specifically, we have included description of the GER previously described to non-mitotically regenerate supporting cells in the abstract, introduction, and discussion. We have also revised the results and discussion sections to point out the differences in the degree of damage and types of supporting cells ablated, leading to differences in the degree of proliferation and the overall cochlear function. We have also included new data using *Plp1^CreERT/+^*; *Rosa26R^DTA/+^*; *Rosa26R^tdTomato/+^* (*Plp1-DTA-tdTomato*) mice (previously used by Mellado Lagarde et al.), where minimal proliferation was observed (Figure S4). These data should help highlight the difference in damage-response observed.

2) I don't find the Connexin26 staining in Figure 4K very convincing. The staining in the inner phalangeal region looks like background especially when compared to the Connexin26 staining at P7 in Figure 3C-D. I also don't understand what is meant by the "plaque-like expression pattern" (page 18)?

Thank you for point this out. We have now removed the data on connexin26 to avoid confusion.

3) I don't understand the point of the Sox2-CreER fate-mapping experiments.  It does not add anything new to the paper that isn't gleaned from fate-mapping with the GLAST-CreER line. Figure 2 showed that the EdU+ cells are in the GER  before the inner phalangeal cells are regenerated, so that's the lead in to using GLAST-CreER for the fate-mapping.  Also there is the confounding factor of Sox2 haploinsufficiency in the Sox2-CreER line that could effect the proliferation seen after Lgr5+ cells are ablated.

All excellent points. We have now removed the Sox2CreER data.

Minor comments

1) Abstract: Why is the Egr1 gene highlighted in the Abstract and Introduction? There were hundreds of genes changed after ablation of Lgr5+ cells so it sees odd to mention just 1 gene in these 2 locations. What makes it special?
We have removed the description of Egr1 from the Abstract and Introduction.

2) Results bottom of page 11: When introducing the Lgr5-DTR mouse, please state that it is a knockin where the endogenous Lgr5 regulatory elements control expression of eGFP and DTR. Similarly when mentioning the Lgr5-CreER line a few sentences later, please state that it is a knockin line and eGFP is the reporter that you refer to (not just "reporter").
We have added these descriptions to the manuscript. (Page 4)

3) Figure 1A would be improved if the GFP channel was also shown alone.
We have added GFP only images to Figure 1A.

4) Figure 1G', I' and K' shows that there is outer HC loss too but this is not mentioned in the Results section until much later in the paper. If you are going to show these images in Figure 1, the HC loss needs to be mentioned here too.
We appreciate the reviewer’s comment and added a description about the loss of OHCs in the result section. (Page 6)

5) Results top of page 13: Figure 1L shows that inner phalangeal cell counts are similar in control and DTR samples at P7, P14 and P21 but P7 was omitted here.

We have now added description of P7 (Page 6, line 2)

6) Results middle of page 13: please clarify that EdU labels cells that are actively dividing when the EdU injections are given, whereas the Ki67 labels cells outside of G0 at the time of sample fixation.
Per reviewer’s suggestion, we have added this description for Ki67 and EdU: “EdU labels actively dividing cells when EdU injections are given and serves as a mitotic tracer, whereas Ki67 labels non-quiescent cells outside G_0_ at the time of sample fixation.” (Page 6, line 20-21)

7) Results top of page 16: The authors conclude that the regenerated cells are inner phalangeal cells based on expression of pannexin1, jagged1 and connexin26. However these 3 genes are also expressed in the GER so I don't think this conclusion is accurate.
We appreciate these comments. We have removed the data on pannexin1, connexin26 and jagged 1 since they are not specific to inner phalangeal cells as pointed out. We have instead added new data showing that regenerated cells express the early inner phalangeal cell marker Fabp7 to figure 3. We have also shown that fate-mapped, regenerated cells express the inner phalangeal cell markers Fabp7 and Glast, in figure 3 and 5. These data support the conclusion that regenerated cell are inner phalangeal cell-like.

8) Figure 4E and 4G would be improved if the myo7a/Vglut3 channel was not present or additional panels without these were added. With the max projections presented as is, it looks like the GLAST/NA-K ATPase staining is surrounding the inner HCs.

We have now included single channel images for Na/KATPα1 and Glast to more clearly show that inner phalangeal cells express these mature markers in figure 5 (previously figure 4).

9) Figure 4P-Q: Need to add a graph of the synapse counts to show the significant differences.
We have added a graph of synapse to Figure 5T (previously figure 4).

10) Results top of page 19: In addition to what is written as a conclusion of this paragraph, the data also shows that ablation of Lgr5+ cells caused synapse loss in inner HCs and loss of outer HCs, resulting in profound hearing loss.

We thank the reviewer for this comment and have revised as follows: “In addition, ablation of Lgr5^+^ supporting cells caused synapse loss in inner HCs and loss of outer HCs, resulting in profound hearing loss. (Page 14, line 1-2)”

11) Results middle of page 20: Please clarify how the expression pattern of GLAST-CreER differs from the Lgr5-DTR expression pattern so that it is clear what cells are fate-mapped vs. killed.  Also please clarify what percentage of GER cells are targeted by GLAST-CreER with the P1 tamoxifen injection.  Finally, Mellado Lagarde et al., 2014 and McGovern et al.., 2019 described the GLAST-CreER expression pattern with tamoxifen given at similar age, please add these citations.
We have revised the text to more clearly describe the expression pattern of GLAST-CreER in the results section and Figure 3 legend (previously figure 5), and how it differs from the Lgr5-DTR expression. We also quantified the percent GER cells that expressed tdTomato after P1 tamoxifen injection (34-38% for damage cochlea and 46-54% for undamaged cochlea at P3) (Page 13, added to Table S7). These results are consistent with those of Mellado Lagarde et al., 2014 and McGovern et al., 2019, which are both cited (Page 9, line 3-4).

12) Results middle of page 21 and Figure 6A-D: The Sparcl1 data seems out of place. Seems like it would fit better in Figure 1 or could be removed if you prefer. Just seems a weird fit for the translatomic figure. Also the GLAST-Cre expression pattern + Edu was just presented in Figure 5 - why is it shown again here?

We have now moved the Sparc1 data to Figure 1. In the previous figures, only P3, P7, P14 and P21 images were shown. Figure 6 (previously Figure 5) shows images from P4 when translatomes were analyzed.

13) Results page 22 & 23: What is nCounter measuring? Transcript level, protein levels, cells? And from what dataset - the RNAscope or something else? In Figure 6I-J and 7L, the nCounter data is presented as normalized counts. How/what is this normalized too?  Details about nCounter and normalization need to be better explained and also added to the methods section.

We thank the reviewer for the chance to clarify what the nCounter is measuring. Briefly, nCounter technology from nanoString measures gene expression at the RNA level. The samples used for this analysis were the same as those used for the RiboTag. We have added a more in-depth description to the methods section and we apologize for the oversight.

14) Results top of page 24: cluster 2 genes are markers of HCs.  This seems odd to me since the gene expression analysis was done using GLAST+ SCs and there is no evidence of HC regeneration after the Lgr5+ cells were ablated. Please add further explanation/speculation in the Discussion section for this.
Cluster 2 genes were highly expressed in Input samples relative to IP samples. Input samples were collected from whole cochleae, prior to RiboTag immunoprecipitation enriching for GLAST-Cre^+^ supporting cells, and as such include hair cells and other non GLAST-Cre^+^ cell populations. We have revised the discussion section to describe this more clearly (p. 22 L6-9).

15) There are several references to neonatal HC regeneration throughout the paper where Cox et al., 2014 is cited but another paper was published at the same time with similar findings (Bramhall et al., 2014) and should also be cited.
We appreciate the reviewer’s information and have added the citation of Bramhall et al., 2014.

16) Discussion: Figure 4P-Q shows a loss of ribbon synapses at P21 in the Lgr5-DTR model and profound hearing loss by ABR and DPOAE in supplemental figures.  However, Mellado Lagarde et al., 2014 saw no change in synapses and no hearing loss when inner phalangeal cells were killed using the PlpCre-DTA model.  These differences should be addressed in the discussion.

This is likely because the Lgr5-DTR model (at 4ng/g) is more damaged that the Plp1-DTA model described by Mellado Lagarde et al. We have added this description to the discussion section (p.19).

17) Figure 2 legend: panels D' and E" are not orthogonal images just images of Edu and Ki67 without Sox2
We apologize for this oversight and have revised the text. (Page 34)

18) Figure 3 legend: CD44 does not label Hensen cells. It labels Claudius cells and LER cells lateral to the Claudius cells (See Chrysostomou et al, 2020).
We appreciate the reviewer’s comment and have revised the description about CD44 labeling. CD44 images have been moved to Supp Fig 5 (previously Figure 3).

19) Figure 4 legend: For A, please add the age tamoxifen was injected as this Tomato pattern will change when TAM is injected at different ages.
We appreciate the reviewer’s comment and have added the tamoxifen injection age in Figure 3 legend. (Page 35).

Reviewer #3:

The sensory epithelium of the mammalian cochlea contains sensory hair cells (HCs) and a variety of supporting cells (SCs). These cells exit the cell cycle during embryonic development and there is very limited capacity for cell regeneration after damage, except for a brief period after birth when both HCs and SCs can be regenerated. While most studies focus on HC regeneration, in this manuscript the authors focus on regeneration of SCs. The authors present a comprehensive study that provides new information on the regenerative capacity of inner ear cells; the findings are novel, advancing the field and the transcriptomic analysis forms the basis to explore the molecular mechanisms underlying this process.

The authors show that after genetic ablation of a subpopulation of SCs, cells in the greater epithelial ridge (GER) re-enter the cell cycle and replace medially located SCs (inner phalangeal cells: iPhCs), but not lateral SCs. They confirm the location and source of progenitors using lineage tracing together with cell ablation. They then characterise the regenerated iPhCs using molecular markers indicating that new cells are integrated in an interconnected network and assessing spontaneous calcium transients, as well as consequences on HC innervation. Overall, their results are consistent with regeneration of iPhCs from GER cells, although the regenerated cells do not fully resemble normal iPhCs. Finally, the authors use the RiboTag mouse to characterise the transcriptional profile of damaged and regenerated cells in comparison to controls and verify a number of differentially expressed and damage/regeneration response genes.

The authors' conclusions are generally well supported by the data presented including the supplementary information. However, the most results appear to be collected from the apex of the cochlea; only a few experiments evaluate mid- or basal turns. This is important since cells in the apex are the last to differentiate. The authors should show data from all three regions throughout all experiments, as customary in the field. If the regenerative capacity is indeed restricted to the apex, the findings cannot be generalised for the entire cochlea as suggested by the authors.
We thank the reviewer for making this important point. We previously presented mainly images from the apical turn in the main figures, and images from middle and basal turns in supplementary figures, even though proliferation and regeneration were observed in all three cochlear turns.

To more clearly show that the proliferation and regeneration of inner phalangeal cells occurred in all three cochlear turns, we have revised main and supplementary figures with graphs and/or images of all 3 turns (Figure 1O-P, 2B-O, 3I, O, 5K-L, Figure S2, S3). These data are also shown in supplementary tables.

I suggest that the authors should reorganise the text and discuss their lineage tracing experiments before the characterisation of regenerated iPhCs. Page 5 of the main text, line 17 the authors conclude: "Collectively, these results indicate that … damage-activated proliferative cells migrate laterally to replace lost IPhCs." This conclusion is not justified by the data presented so far, but can be substantiated by the lineage tracing experiments.
We thank this reviewer for this comment. We have reorganized the manuscript to now include the lineage tracing data earlier in the results section as Figure 3 (previously Figure 5). Furthermore, we have added new data showing that Glast-Cre^+^ fate-mapped cells expressed the inner phalangeal cell marker, Fabp7, after migrating to the inner phalangeal cell region (Figure 3). We have also revised the results section to more clearly describe these findings and how they support the conclusion that “damage-activated proliferative cells migrate laterally to replace lost IPhCs”.
