# Supplementary material for: Lineage-tracing and translatomic analysis of damage-inducible mitotic cochlear progenitors identifies candidate genes regulating regeneration
Source: PLoS Biol. 2021 Nov 10;19(11):e3001445. doi: 10.1371/journal.pbio.3001445 (PMC8608324; doi:10.1371/journal.pbio.3001445)
Supplement: Attachment — Submitted filename: Pbio point by pointR2V2.docx [file pbio.3001445.s028.docx]

Reviewer #1:

This is a revision for the resubmission of a manuscript on the regenerative potential of an Lgr5-independent cell population in the GER. By disrupting the Lgr5 population, they show that the GER houses competent cells. 

From the perspective of regeneration, the findings in the paper are not new. The study includes translatome analyses, but the manuscript does little with this information to highlight any mechanistic understanding, which is what is lacking in the field. The study does not clearly distinguish between damage or regeneration responses. The way sections are written (abstract, introduction), the reader is misled to think that the differentially expressed genes are associated with regeneration until they get to the discussion, which is a little late. The authors will want to consider approaches to overexpress candidate genes for regeneration to support the regeneration pitch of this manuscript.

We thank this reviewer for this feedback. Per the editor’s recommendation and to clarify our findings, we have changed the title to “Lineage-tracing and translatomic analysis of damage inducible mitotic cochlear progenitors identifies candidate genes regulating regeneration”. We have also planned future experiments to overexpress candidate genes for regeneration.

Despite CtBP2 synapses being drastically (quantitatively too) reduced, in the response letter, the authors claim that the IHC remain innervated? I'm not sure how that conclusion was reached.

This is a good point. We have relied on the literature describing synaptopathy, where hair cells with synaptopathy and co-localized pre- and post-synapses are described as innervated (Kujawa and Liberman 2015, Liberman and Kujawa, 2017). Since we did not have TEM or electrophysiological evidence of innervation, we have stated “Vglut3^+^ IHCs… appeared to remain innervated”.

At this point, I cannot agree that the Ca transients are associated with regeneration (as suggested in the abstract). Thus far, most of the data suggest damage responses (despite several pieces of data have been removed), other than the one regeneration phenotype that has been described by several others. Other than ruling out a marker of interest (Lgr5), as mentioned before, it has been a long-known that the GER holds such a potential.

Calcium transients were observed at P7, an age when regeneration of inner phalangeal cells was observed. This observation supports the statement that regenerated supporting cells display coordinated calcium transients in the abstract.

The authors need to carefully interpret or reevaluate their narration and conclusions, as it will cause a lot of confusion.

Thank you for this feedback. We have carefully interpreted and re-evaluated our narration and conclusions.

Reviewer #2:

Thank you for the thorough response to the reviewer comments. The manuscript is much improved and now ready for publication.

Thank you.

Reviewer #3:

The authors have addressed previous comments and improved the manuscript.

Thank you.
